# Thoracic Spinal Arachnoid Cyst in a Patient With Cerebral Palsy: Clinical Evaluation and Technical Note

**DOI:** 10.7759/cureus.93917

**Published:** 2025-10-06

**Authors:** Jordan M Rasmussen, Patrick Opperman, Afshin Salehi

**Affiliations:** 1 Neurosurgery, University of Nebraska Medical Center, Omaha, USA

**Keywords:** cerebral palsy, flexible endoscope, laminectomy, spasticity, spinal arachnoid cyst

## Abstract

Spinal arachnoid cysts (SACs) are uncommon lesions that may lead to neurological decline due to spinal cord compression. They are particularly rare in the pediatric population and even more so among patients with cerebral palsy (CP). We present a case of a four-year-old female with CP undergoing evaluation for spasticity in the context of potential candidacy for selective dorsal rhizotomy. Imaging revealed a dorsal thoracic SAC extending from the level of T4 to T9, causing significant spinal cord compression. Given that differentiating spasticity-associated symptoms attributable to CP versus the arachnoid cyst was difficult, surgical intervention was pursued prior to rhizotomy consideration. The patient underwent a two-level thoracic laminectomy, consisting of a total laminectomy at T6 and a partial laminectomy involving the superior aspect of T7, with endoscopic-assisted cyst fenestration. Her postoperative course was uneventful, and follow-up myelography confirmed reestablished cerebrospinal fluid (CSF) flow and spinal cord decompression. This case highlights the importance of thorough evaluation when managing spasticity in pediatric patients with CP, as well as the utility of flexible endoscopic-assisted fenestration as a minimally invasive treatment option for SACs.

## Introduction

Spinal arachnoid cysts (SACs) are cerebrospinal fluid (CSF)-filled lesions that can compress the spinal cord and cause progressive neurological deficits. They are typically seen in adult males, often in the thoracic spine, but can also occur in pediatric populations, albeit rarely [1-3]. SACs may be congenital or acquired, with secondary causes including trauma, infection, or prior CSF diversion procedures [4,5]. They can present with a heterogeneous collection of symptoms. In children, this can include lower extremity weakness and urinary continence issues. Both symptoms can be confounders in a patient with CP.

Cerebral palsy (CP) refers to a group of non-progressive neurodevelopmental disorders resulting from disturbances in brain development. Motor impairments such as spasticity, dyskinesia, and ataxia are common [6,7]. Spastic diplegia is one of the most frequent CP motor phenotypes. Although CP itself is considered static, the emergence of new or worsening neurological symptoms should prompt evaluation for other causes [8]. Spinal lesions, including SACs, can exacerbate or mimic spasticity, complicating diagnosis [8,9].

The use of spinal imaging in CP patients being evaluated for rhizotomy remains debated. However, recent studies suggest its utility in ruling out structural lesions that could worsen spasticity or influence surgical risk [9-11]. Here, we describe a pediatric CP patient with a complicated history who was undergoing surgical evaluation for spasticity and was found to have a SAC.

## Case presentation

A four-year-old female with spastic diplegic CP was referred for surgical evaluation in the management of her spasticity. She had no history of trauma but did have a history of ventriculoperitoneal shunt complicated by infection and need for external ventricular drain (EVD) within her first couple of years of life. As part of her surgical evaluation for rhizotomy, she underwent lumbar spine imaging. This revealed the inferior edge of a spinal canal lesion necessitating further imaging of the spinal axis.

**Figure 1:**
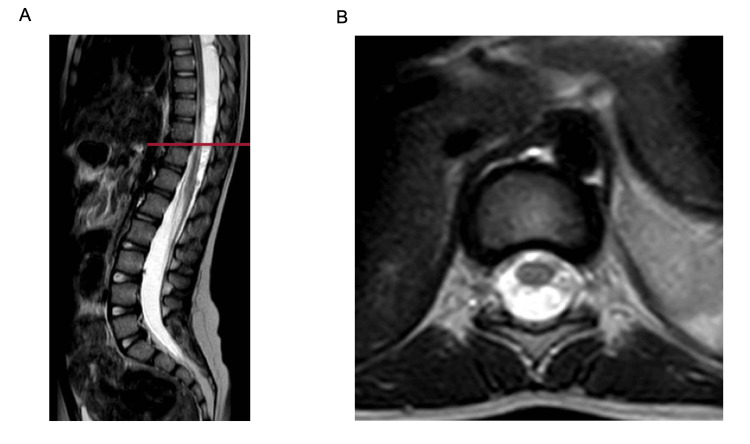
Sagittal (A) and axial (B) T2-weighted image from the initial MRI lumbar spine for preliminary evaluation, showing a dorsal CSF-intensity lesion causing ventral displacement and compression of the thoracic spinal cord.

**Figure 2:**
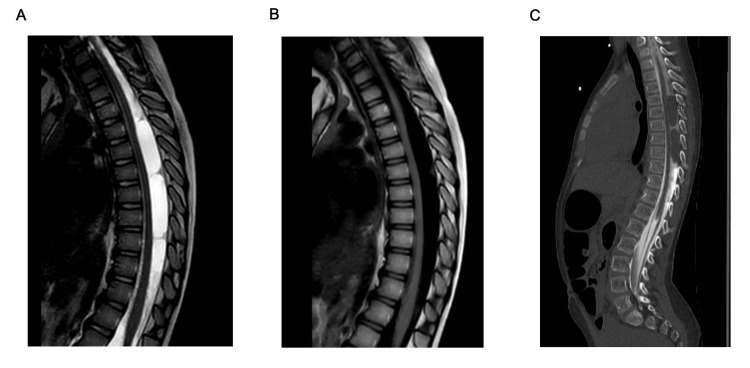
Sagittal MRI thoracic spine, sagittal T2 (A) and T2 flair (B), demonstrate imaging characteristics consistent with cerebrospinal fluid. CT myelogram (C) images showing interruption of normal contrast flow at the mid-thoracic level due to mass effect from the arachnoid cysts.

T2-weighted sagittal MRI of the spinal axis revealed dorsal fluid collections from T4 to T9, causing ventral spinal cord displacement and compression (Figure 1). Follow-up myelography demonstrated two non-enhancing intradural cystic lesions within the dorsal thoracic thecal sac, measuring 1.1 x 1.6 x 3.3 cm and 1.1 x 1.4 x 3.7 cm, respectively, both producing significant spinal cord compression (Figure 2). The presence of SAC and compression of the thoracic spinal cord complicated the evaluation of her spasticity and potential need for rhizotomy; therefore, we decided it was best for her to undergo fenestration of the cysts prior to further consideration of rhizotomy. 

**Figure 3:**
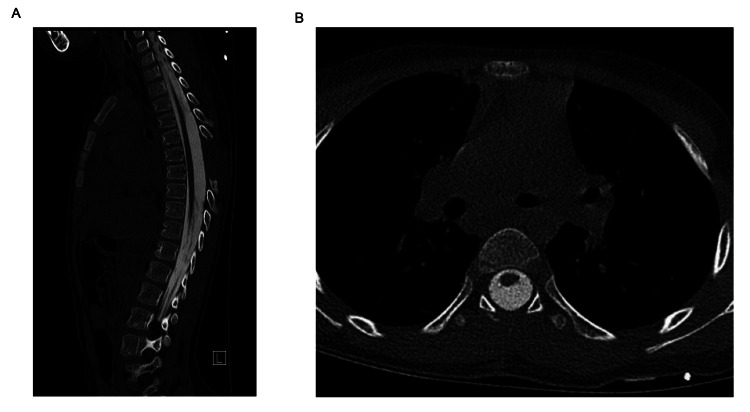
Sagittal (A) and axial (B) myelogram images after fenestration showing reconstitution of contrast flow and resolution of spinal cord displacement.

The size of the SAC expanded to six thoracic levels. After careful consideration, the patient underwent a two-level thoracic laminectomy, consisting of a complete laminectomy at T6 and a partial laminectomy involving the superior aspect of T7, with flexible endoscopic-assisted fenestration of the SAC. Intraoperative fluoroscopy was used for the localization of the limited laminectomy at the adjoining level of the two cysts. A flexible endoscope was selected to enhance visualization rostrally and caudally to minimize the levels needed for a laminectomy. This was felt to be safe given the ventral displacement of the thoracic cord and the presence of sufficient room dorsally for the maneuverability of the flexible endoscope. Her postoperative recovery was uncomplicated, with no new neurological deficits. Follow-up myelography showed improved CSF flow and relief of spinal cord compression (Figure 3). After further evaluation and discussion with our spasticity team, she later underwent bilateral selective dorsal rhizotomy, combined with ventral rhizotomy. Several months later, she continues to demonstrate improvements in her mobility with improvement of her spasticity.

## Discussion

SACs are rare but clinically significant lesions that are often asymptomatic, but may present with pain, sensory changes, weakness, myelopathy, radiculopathy, bladder dysfunction, or spasticity. In our clinical scenario, this patient’s SAC was identified as part of their surgical evaluation for spasticity. The dorsal thoracic location and multilevel extension seen in this case align with common SAC patterns described in the literature [3,12]. SAC usually presents as a solitary lesion, but it is possible to see multiloculated or septated arachnoid cysts, as is the case with our patient.

The exact cause of SACs in pediatric patients is often unclear. However, congenital and acquired factors are both implicated. Prior CSF diversion or infection increases the risk of secondary SAC formation, likely due to arachnoid inflammation and subsequent CSF loculation [3,5,10]. Congenital theories are often considered when there is no preceding history to suggest an acquired pathology. Our patient’s history of CP and early life VP shunt followed by CSF infection necessitating external ventricular drainage suggests the possibility of an acquired etiology. However, the definitive etiology is difficult to discern in this situation.

As part of the evaluation for spasticity, one must consider spinal pathology and imaging in this workup. This may be confounded in patients with a history of CP and known spastic diplegia. However, it is documented that SAC patients who undergo intervention and surgical treatment have a strong likelihood of improvement in symptoms related to the SAC. Recent reports recommend spinal imaging in patients with atypical or progressive symptoms being evaluated for rhizotomy to rule out structural abnormalities that may alter management [9-11]. This case reinforces that recommendation.

**Figure 4:**
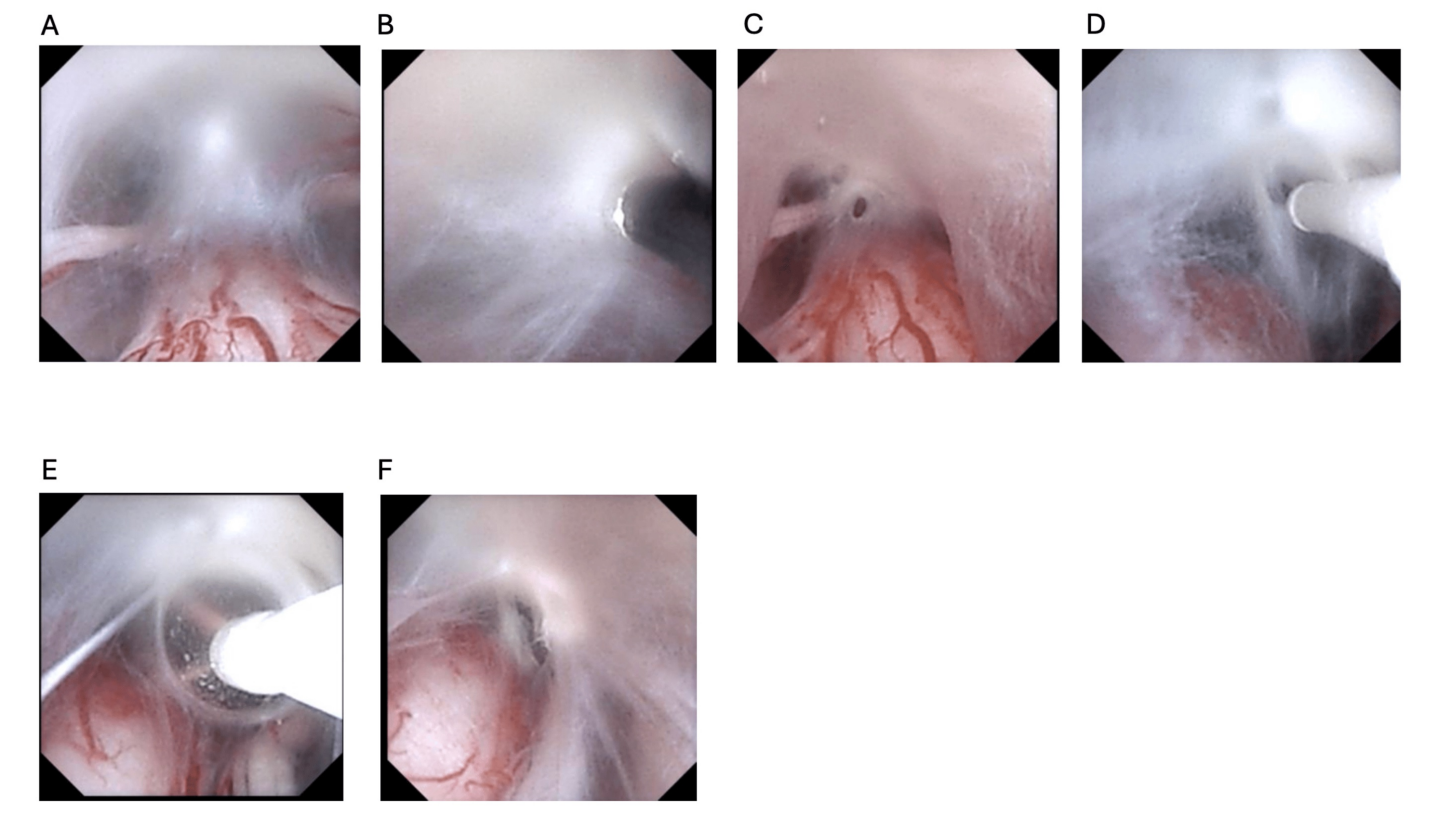
Images demonstrate the intradural viewpoint working through the endoscope in stepwise fashion from initial view through successive enlargement of the fenestration for continuity (A-F).

Surgical options for symptomatic SACs include fenestration, partial or complete excision, and, in select cases, cystoperitoneal shunting. Population-based cohort studies suggest that while both fenestration and resection can relieve symptoms, complete resection tends to offer more durable results and lower recurrence rates long term [10,13-16]. Endoscopic-assisted fenestration, as performed here, provides a minimally invasive approach that may reduce morbidity compared to open techniques [11,16,17]. Flexible endoscope-assisted fenestration of the arachnoid cyst can help limit the levels of laminectomy. In addition, one must ensure that there is sufficient space within the spinal canal - intradural - to facilitate the use of the endoscope without undue pressure on the spinal cord. Lastly, it is important to navigate the endoscope through the terminal end of the cyst to visualize the subarachnoid space to ensure free flow of CSF without evidence of additional pathology (Figure 4) [18-20].

In this situation, a traditional multilevel laminectomy and durotomy for excision of the arachnoid cysts was felt to be morbid and would likely necessitate future fusion. The use of a flexible endoscope with a limited two-level laminectomy allowed fenestration of the extensive arachnoid cysts, and it was felt to be a reasonable option that should be considered for future patients. While our patient’s outcome was favorable, recurrence rates are higher with fenestration than with complete excision, making long-term follow-up essential [3,13,14,16].

## Conclusions

SACs, although rare, should be considered in CP patients with new or worsening spasticity. These lesions can mimic or exacerbate motor symptoms and may be reversible with appropriate treatment. MRI and myelography are critical for diagnosis and surgical planning. Endoscopic-assisted fenestration is a safe, effective option for symptomatic SACs, but careful long-term monitoring is warranted to detect potential recurrence.
